# Cerebrospinal fluid β2-microglobulin as a diagnostic biomarker in central nervous system lymphoma: a single-center retrospective analysis

**DOI:** 10.1007/s00277-025-06719-x

**Published:** 2025-11-10

**Authors:** Yijun Fan, Yuyang Huang, Wenwen Zheng, Zhouzhou Su, Jun Hu, Shunzong Yuan

**Affiliations:** 1 Senior Department of Hematology, Chinese PLA General Hospital, Beijing, 100071 China; 2 Medical School of Chinese PLA, Beijing, 100853 China; 3 Department of Hematology, State Key Laboratory of Complex Severe and Rare Diseases, Peking Union Medical College Hospital, Chinese Academy of Medical Sciences and Peking Union Medical College, Beijing, 100730 China; 4 Department of Laboratory Medicine, The Fifth Medical Center of PLA General Hospital, Beijing, 100071 China; 5 Programme Committee for the Study Programmes in Biomedicine, Karolinska Institute, Stockholm, 17177 Sweden; 6 Department of Neurology, Southwest Hospital, Third Military Medical University (Army Medical University), Chongqing, 400038 China

**Keywords:** Central nervous system lymphoma, Β2-microglobulin, Cerebrospinal fluid, Diagnostic biomarker

## Abstract

This retrospective cohort study investigated the diagnostic performance of cerebrospinal fluid (CSF) β2-microglobulin (β2-M) in patients with central nervous system lymphoma (CNSL). Between January 2018 and August 2024, 1,349 hospitalized patients with CSF β2-M in our center were categorized into lymphoma, leukemia, solid tumor, and other disease cohorts, with additional stratification by central nervous system involvement. CSF β2-M concentrations were markedly elevated in CNSL relative to all other comparator groups (*p* < 0.001). A cut-off value of 1.85 mg/L discriminated CNSL from non-CNS-involved lymphoma with high diagnostic accuracy, yielding 85.7% sensitivity and 89.7% specificity. Longitudinal assessment further demonstrated that dynamic CSF β2-M trajectories correlated with therapeutic response and relapse risk. Collectively, these findings establish CSF β2-microglobulin as a reliable, accessible, and cost-effective diagnostic biomarker for CNSL.

## Introduction

Central nervous system lymphoma (CNSL) is a rare but aggressive subtype of extranodal non-Hodgkin lymphoma, accounting for 3 to 4% of all CNS tumors [1]. CNSL can be divided into primary and secondary CNSL (PCNSL and SCNSL) based on whether tumor cells involve the brain, eyes, meninges, or spinal cord without or with evidence of systemic disease. For SCNSL, the diagnosis can be easily achieved by combining non-brain biopsy pathology and radiographic imaging patterns. However, for most PCNSL, the only way to obtain a definitive diagnosis is histopathological confirmation after invasive brain biopsy [1]. Deep-seated lesions, in particular, often preclude safe sampling, and their invasiveness carries substantial risks, including intracranial hemorrhage, cerebral edema, and seizures [2]. Although a positive finding in other samples, such as cerebrospinal fluid (CSF) and vitreous biopsy, can to some extent avoid the need for a surgical procedure, a safer and less invasive diagnostic method for CNSL has yet to be established. CSF assessment and intrathecal chemotherapy are routine for CNSL in some centers [3]. Several outstanding studies have demonstrated that interleukin-10 (IL-10), the C-X-C motif chemokine ligand 13 (CXCL13), and circulating tumor DNA (ctDNA) in CSF are reliable markers for adjuvant diagnosis of CNSL, but lack universal applicability due to methodological complexity or inconsistent diagnostic thresholds [4, 5]. Consequently, a widely suitable biomarker in the CSF for the diagnosis of patients with CNSL is still an unmet need [6].

β2-microglobulin (β2-M), an 11.8-kDa subunit of major histocompatibility complex class I (MHC-I) molecules, is detectable in multiple body fluids, including CSF, serum, and urine [7]. Elevated CSF β2-M levels are consistently observed in diverse CNS pathologies, including neoplastic, inflammatory, and neuroimmune disorders, with particularly marked elevations in CNSL [4, 8–10]. Mavligit et al. [11] first identified elevated CSF β2-M with a mean level of 4.2 ± 0.6 mg/L in CNSL patients. Recent studies proposed CSF β2-M cut-offs at 2.4 mg/L (CNSL vs. tumors) [12] and 2.056 mg/L (PCNSL vs. other CNS malignancies/inflammation) [13] for differential diagnosis. However, previous findings are limited by small cohort sizes and a lack of validation across heterogeneous CNS diseases. To address these gaps, we conducted a retrospective study to assess the diagnostic value of CSF β2-M in CNSL in this single-center study.

## Methods

### Study population

A total of 1,349 patients with confirmed or suspected central nervous system (CNS) disease were studied at the Fifth Medical Center of the People’s Liberation Army General Hospital between January 2018 and August 2024. The analysis was restricted to patients with first-onset disease, initial management, and availability of concurrent CSF and serum β2-M measurements; those with uncertain diagnoses or incomplete medical records were excluded. This study was approved by the medical ethics review committee of our hospital (KY-2025-10-187-1).

**Fig. 1:**
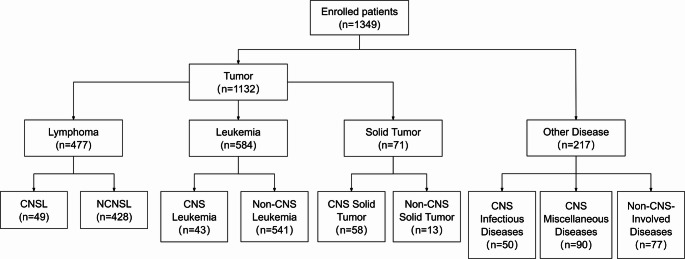
Patient enrollment and stratification. CNSL, central nervous system lymphoma; NCNSL, non-CNS lymphoma

Based on diagnoses defined by World Health Organization (WHO) criteria, patients were stratified into four major cohorts: lymphoma, leukemia, solid tumor, and other non-neoplastic diseases (Fig. 1). Within the lymphoma cohort, patients were further classified based on CNS involvement into CNSL and non-CNS lymphoma (NCNSL), with CNSL including diffuse large B-cell lymphoma (DLBCL, *n* = 41), NK/T-cell lymphoma (*n* = 2), peripheral T-cell lymphoma (*n* = 3), Burkitt lymphoma (*n* = 2), and unclassified B-cell lymphoma (*n* = 1). Similarly, in the leukemia cohort, patients were divided into CNS leukemia and non-CNS leukemia. The solid tumor cohort contained CNS solid tumors and non-CNS solid tumors, with CNS solid tumors comprising gliomas (*n* = 2), meningioma (*n* = 2), and lung (*n* = 32), spine (*n* = 1), cervix (*n* = 1), breast (*n* = 16), or gastric (*n* = 4) cancer metastases. Other disease cohorts included CNS infectious diseases, CNS miscellaneous diseases, and non-CNS-involved diseases. CNS miscellaneous diseases group included cerebrovascular disease (*n* = 41), demyelinating disease (*n* = 8), consciousness disorders (*n* = 12), epilepsy (*n* = 13), neuroimmune disease (*n* = 13), and toxic encephalopathy (*n* = 3).

### Sample measurement

β2-M concentrations in CSF and serum were measured using a latex-enhanced immunoturbidimetric assay with a commercial reagent kit (Zybio, Chongqing, China) on HITACHI Labospect 008AS or HITACHI 7600 − 110 analyzers (Tokyo, Japan). The assay employed an endpoint method read at 600 nm. All measurements were performed on the day of sample collection or within 72 h, with samples stored at 2–8 °C. The analytical measuring range for β2-M was 0.30–20.00 mg/L. For clinical interpretation, the manufacturer’s recommended reference interval (0.8–2.8 mg/L) was applied to serum β2-M levels, while CSF β2-M levels were referenced against the established interval of 0.6–2.0 mg/L [14]. Concurrently, total protein and glucose levels in CSF were assessed using standard clinical chemistry packages on the same platforms. CSF IL-10 levels were determined using a commercial chemiluminescence immunoassay kit (Siemens Healthcare Diagnostics Products Ltd., Gwynedd, UK) on an Immulite 1000 Analyzer (Siemens Healthcare Diagnostics Inc., NJ, USA), with a reported measurement range of 5–1000 pg/mL.

### Statistical analysis

All statistical analyses were conducted using GraphPad Prism software (version 10.0). Continuous variables are presented as median (range). Comparisons between two groups were performed using the Mann–Whitney U test or chi-square test, while the Kruskal–Wallis test was used for comparisons across multiple groups. The relationship between CSF β2-M and serum β2-M levels was assessed by Spearman’s rank correlation. For post hoc pairwise comparisons, the Bonferroni correction was applied using the Dunn test function from the FSA package. Diagnostic performance was assessed by calculating the area under the receiver operating characteristic (ROC) curve. A two-sided p-value of less than 0.05 was considered statistically significant.

## Results

### Utility of CSF β2-M as a CNSL biomarker

**Fig. 2:**
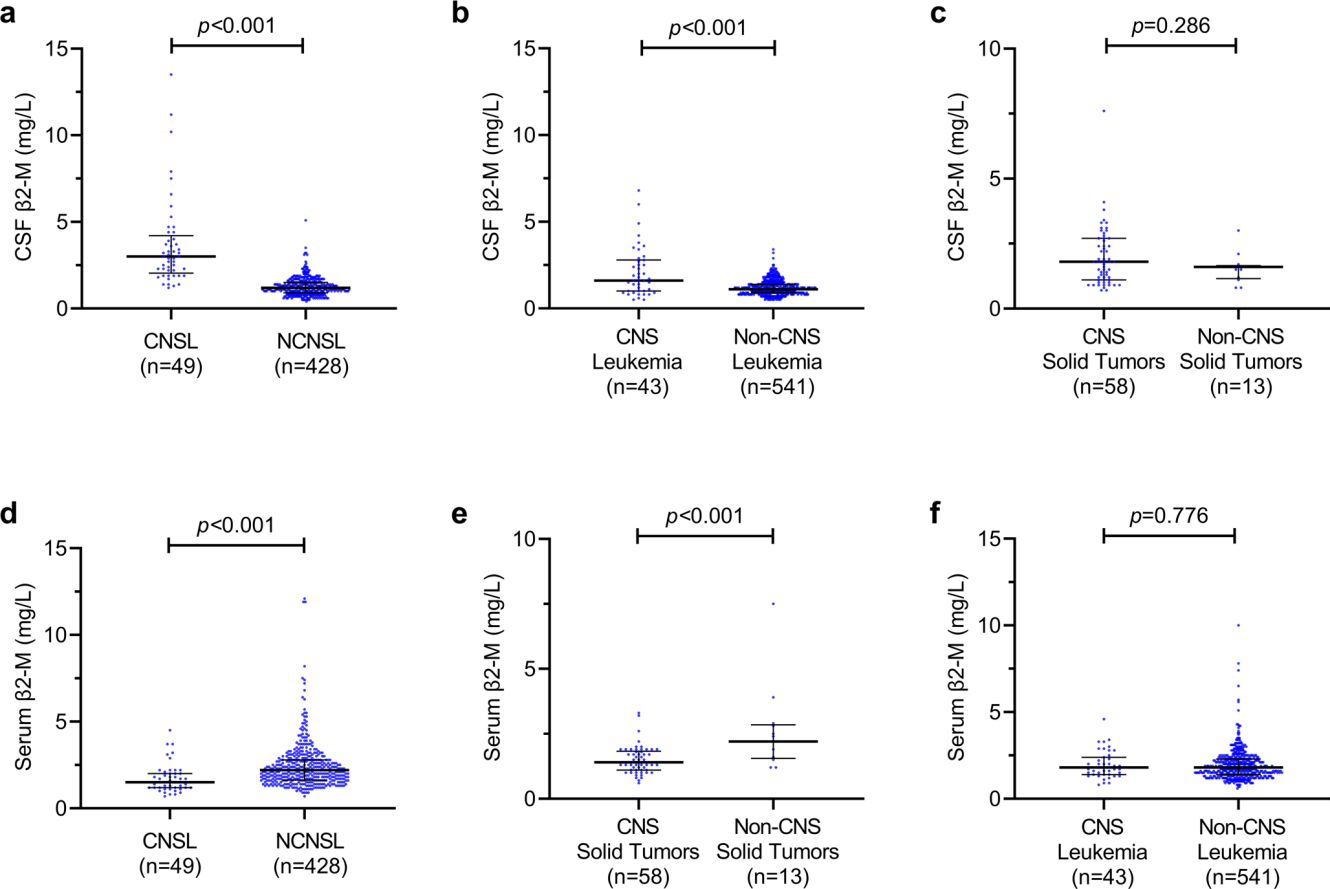
Comparison of β2-M levels across different patient groups in CSF and serum, respectively. Comparison of CSF β2-M levels between patients with CNSL and NCNSL (**a**), CNS leukemia and non-CNS leukemia (**b**), CNS solid tumors and non-CNS solid tumors (**c**); Comparison of serum β2-M levels between patients with CNSL and NCNSL (**d**), CNS solid tumors and non-CNS solid tumors (**e**), CNS leukemia and non-CNS leukemia (**f**)

CSF and serum β2-M levels of each group are shown in Table 1. In the CNSL group, the median CSF β2-M level was 3.0 (range, 1.2–13.5) mg/L, significantly higher than other groups (*p* < 0.001 for CNS leukemia, CNS solid tumor, and CNS miscellaneous diseases; *p* = 0.095 for CNS infectious diseases). In contrast, a comparison of CSF total protein levels between CNSL, CNS leukemia, CNS solid tumor, and CNS infectious disease patients revealed no significant difference (*p* = 0.312). Serum β2-M concentrations of CNSL demonstrated considerable overlap across other groups, confirming its inability to discriminate CNSL from other CNS-involved tumors. Critically, patients with CNS involvement of hematopoietic malignancies had significantly higher CSF β2-M levels than those without (CNSL vs. NCNSL: 3.0 mg/L vs. 1.2 mg/L; CNS leukemia vs. non-CNS leukemia: 1.6 mg/L vs. 1.1 mg/L; both *p* < 0.001) (Figs. 2a, b). However, there was no significant difference in β2-M levels between patients with and without solid tumor CNS involvement (CNS solid tumor vs. non-CNS solid tumor: 1.8 mg/L vs. 1.6 mg/L, *p* = 0.286) (Fig. 2c). Furthermore, serum β2-M levels were significantly lower in patients with CNS involvement than in those without lymphoma (CNSL vs. NCNSL: 1.5 mg/L vs. 2.2 mg/L, *p* < 0.001) or solid tumors (CNS solid tumor vs. non-CNS solid tumor: 1.3 mg/L vs. 2.2 mg/L, *p* < 0.001), but serum β2-M levels in patients with CNS leukemia were the same as non-CNS leukemia (1.8 mg/L, *p* = 0.776) (Figs. 2d-f).

**Table 1:** Baseline characteristics and laboratory markers in each groups.^*^

Group	Sex, male/ female, No.(%)	Age, y	CSF TP, g/L	CSF β2-M, mg/L	Serum β2-M, mg/L
CNSL (*n* = 49)	32/17 (65/35)	58 (29, 75)	0.70 (0.27, 5.25)	3.0 (1.2, 13.5)	1.5 (0.7, 4.5)
PCNSL (*n* = 31)	20/11 (65/35)	56 (33, 75)	0.57 (0.27, 2.98)	2.7 (1.3, 13.5)	1.2(0.7, 2.2)
SCNSL (*n* = 18)	12/9 (57/43)	61 (29, 74)	0.84 (0.4, 5.25)	3.2 (1.2, 11.2)	1.8 (1.3, 4.5)
NCNSL (*n* = 428)	268/160 (63/37)	51 (12, 90)	0.36 (0.11, 5.20)	1.2 (0.4, 5.1)	2.2 (0.7, 12.1)
CNS Leukemia (*n* = 43)	27/16 (63/37)	39 (16, 71)	0.61 (0.18, 5.59)	1.6 (0.5, 6.8)	1.8 (0.8, 4.6)
Non-CNS Leukemia (*n* = 541)	309/232 (57/43)	40 (11, 78)	0.35 (0.14, 16.60)	1.1 (0.5, 3.4)	1.8 (0.6, 10.0)
CNS Solid Tumor (*n* = 58)	19/39 (33/67)	52 (31, 75)	0.56 (0.23, 3.75)	1.8 (0.7, 7.6)	1.3 (0.6, 3.3)
Non-CNS Solid Tumor (*n* = 13)	5/8 (38/62)	49 (36, 74)	0.43 (0.13, 3.51)	1.6 (0.8, 3.0)	2.2 (1.2, 7.5)
CNS Infectious Diseases (*n* = 50)	34/16 (68/32)	11 (0, 75)	0.60 (0.15, 5.50)	2.3 (0.3, 21.5)	2.4 (0.7, 8.1)
CNS Miscellaneous Diseases (*n* = 90)	52/38 (58/42)	51 (0, 98)	0.44 (0.10, 2.25)	1.7 (0.3, 7.1)	1.9 (0.6, 24.8)
Non-CNS-Involved Diseases (*n* = 77)	51/26 (34/66)	0 (0, 68)	0.63 (0.13, 3.13)	1.7 (0.5, 5.8)	2.6 (1.1, 5.3)

* Data are presented as median (range)

Abbreviations: CNSL, central nervous system lymphoma; PCNSL, primary CNSL; SCNSL, secondary CNSL; NCNSL, non-CNS lymphoma; TP, total protein

### Diagnostic performance of CSF β2-M in patients with CNSL

**Fig. 3:**
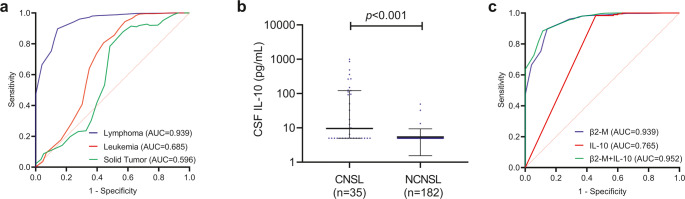
Diagnostic performance of CSF biomarkers for distinguishing CNSL from NCNSL. ROC curve analysis of CSF β2-M in discriminating CNSL from NCNSL, CNS leukemia from non-CNS leukemia, and CNS solid tumor from non-CNS solid tumor (**a**). CSF IL-10 levels in patients with CNSL compared to NCNSL (**b**). ROC analysis comparing the diagnostic accuracy of CSF β2-M, CSF IL-10, and β2-M + IL-10 for distinguishing CNSL from NCNSL (**c**)

ROC analysis found that a CSF β2-M threshold of 1.85 mg/L could distinguish CNSL from NCNSL with an area under the curve (AUC) of 0.939 (*p* < 0.001), sensitivity of 85.7% (42/49), and specificity of 89.7% (384/428), with a positive predictive value 48.8% (42/86) and a negative predictive value 98.2% (384/391). In contrast, CSF β2-M showed lower diagnostic accuracy for differentiating CNS leukemia from non-CNS leukemia (AUC: 0.685, *p* < 0.001) and CNS solid tumor from non-CNS solid tumor (AUC: 0.596, *p* = 0.281), underscoring its specificity for CNSL (Fig. 3a). Due to limited routine clinical use, CSF IL-10 levels were available for only 217 patients. CSF IL-10 levels were significantly elevated in CNSL compared to NCNSL (CNSL vs. NCNSL: 9.62 pg/mL vs. 5 pg/mL, *p* < 0.001) (Fig. 3b). ROC analysis yielded an AUC of 0.765 for CSF IL-10, with a sensitivity of 54.3% and specificity of 98.4% at an optimal cut-off of 5.745 pg/mL. When combined, CSF β2-M and IL-10 improved diagnostic accuracy further, achieving an AUC of 0.952, sensitivity of 91.4%, and specificity of 88.5%. This combined biomarker panel outperformed IL-10 alone in detecting CNSL (*p* < 0.001) (Fig. 3c).

### Correlation between CSF and serum β2-M

**Fig. 4:**
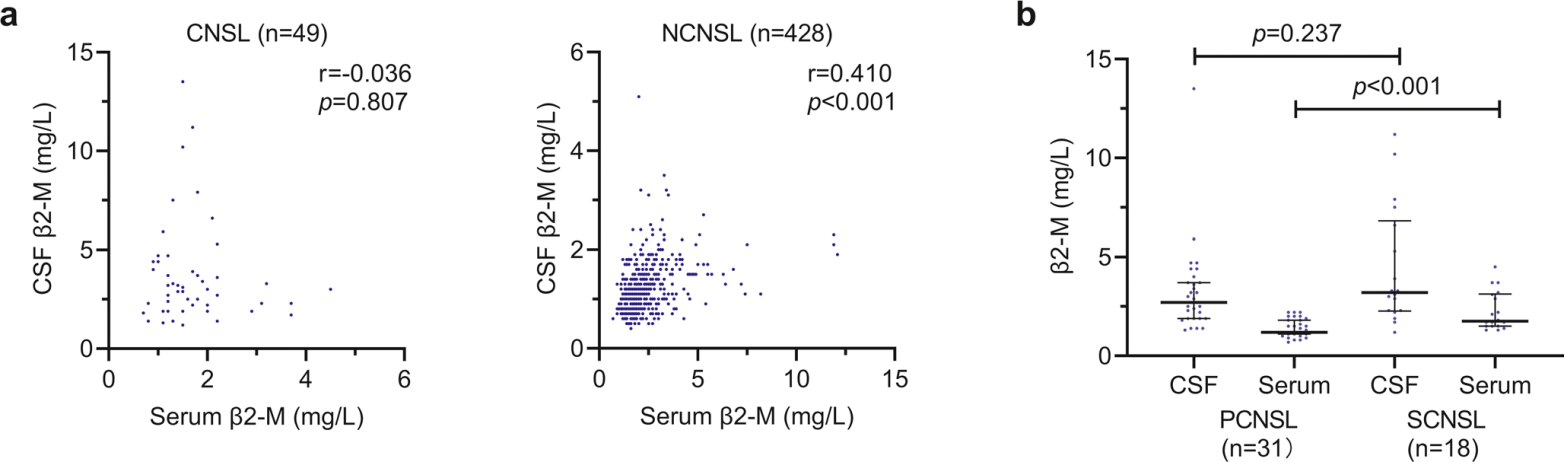
Analysis of CSF and serum β2-M correlation and comparison in patients with CNSL or NCNSL. CSF and serum β2-M correlation analysis in patients with CNSL or NCNSL (**a**). Comparison of CSF and serum β2-M levels in patients with PCNSL and SCNSL (**b**)

While seminal work by Starmans et al. reported dissociation between serum and CSF β2-M levels in neuroinfectious diseases [15], this relationship in CNS malignancies—particularly CNSL—remains largely unexplored. Our data demonstrated that in patients with non-CNS-involved conditions such as NCNSL, non-CNS leukemia, non-CNS solid tumor, and non-CNS-involved disease, β2-M levels were significantly higher in serum than in CSF, and in contrast to patients with CNS involved by lymphoma or solid tumor, β2-M levels were significantly higher in CSF than in serum (Table 1). In NCNSL patients, CSF β2-M levels were significantly lower than those in serum (CSF vs. Serum: 1.2 mg/L vs. 2.2 mg/L, *p* < 0.001), and the two compartments showed a moderate positive correlation (*r* = 0.410, *p* < 0.001). Conversely, in CNSL patients, CSF β2-M levels were double those of the serum levels (CSF vs. Serum: 3.0 mg/L vs. 1.5 mg/L, *p* < 0.001), and this was accompanied by a weak negative correlation between them (*r* = − 0.036, *p* = 0.807) (Fig. 4a). Comparative analysis also showed that PCNSL patients had significantly lower serum β2-M levels than SCNSL patients (PCNSL vs. SCNSL: 1.2 mg/L vs. 1.8 mg/L, *p* < 0.001). However, CSF β2-M levels showed no difference between PCNSL and SCNSL (2.7 mg/L vs. 3.2 mg/L, *p* = 0.237) (Fig. 4b).

### CSF β2-M dynamics indicate treatment efficacy in CNSL

**Fig. 5:**
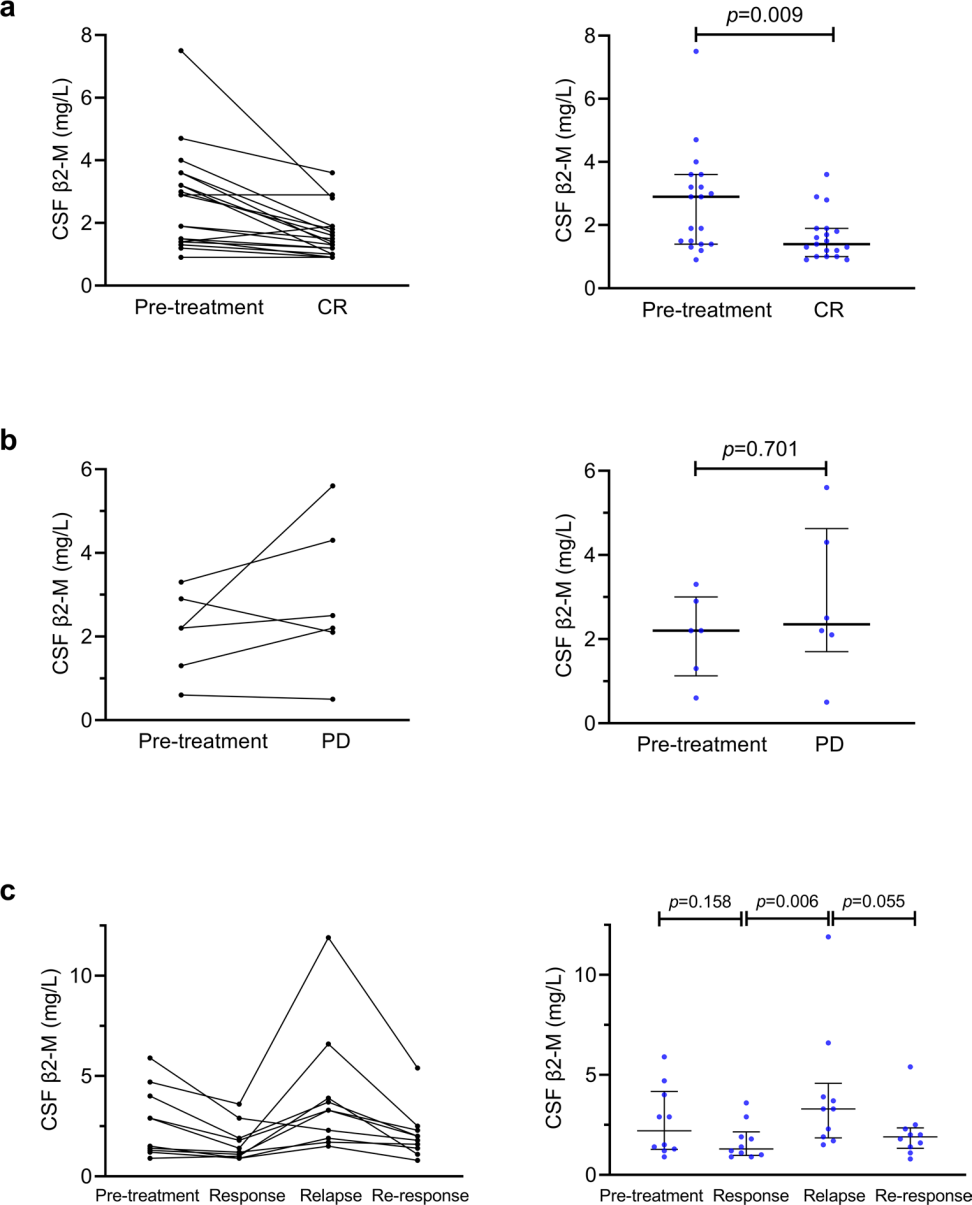
Dynamic CSF β2-M level changes after treatment. CSF β2-M level changes in patients with CNSL treated with first-line therapy are shown before treatment and after treatment in CR (**a**) and PD (**b**). Dynamic CSF β2-M level changes in patients with CNSL treated with first-line and rescue therapies are shown before treatment and in response, relapse, and re-response after treatment (**c**)

All 35 patients diagnosed with CNSL were treated with intrathecal methotrexate chemotherapy after hospital admission. CSF β2-M concentrations were assessed at multiple time-points during the treatment (Figs. 5a-c). In patients with complete remission (CR) (*n* = 19), CSF β2-M levels showed a significant downward trend, with a reduction of nearly half from baseline after treatment (2.9 mg/L vs. 1.4 mg/L, pre-treatment vs. CR, *p* = 0.009). Conversely, progressive disease (PD) (*n* = 6) was associated with persistent or minor elevated levels (2.2 mg/L vs. 2.35 mg/L, pre-treatment vs. PD, *p* = 0.701). Among 10 relapsed patients—3 with partial response (PR) and 7 with CR prior to relapse—CSF β2-M levels significantly increased from remission levels to relapse state (1.3 → 3.3 mg/L, response → relapse, *p* = 0.006), and decreased to 1.9 mg/L after retreatment (*p* = 0.055).

## Discussion

In this study, we demonstrated that CSF β2-M is significantly elevated in CNSL, shows a dynamic “high-low-high” pattern correlating with disease course, and may suggest local production within the CNS. Its diagnostic cut-off aligns with historical reports, affirming reliability across platforms. The diagnostic utility of this biomarker is strengthened by its consistent performance across different technical platforms. Specifically, the diagnostic cut-off established in our larger cohort aligns with, yet refines, historically reported thresholds. These include values such as 1.6 mg/L (microparticle enzyme immunoassay, MEIA) reported by Kersten et al., 1.9 mg/L (radioimmunoassay) by Ernerudh et al., and 1.89 mg/L (enzyme linked immunoassay, ELISA) by Hansen et al. [16–18]. Slight variations in these absolute cut-off values are likely attributable to differences in assay techniques. Reassuringly, however, CSF β2-M concentrations in CNSL patients have been consistently reported within the 2–4 mg/L range across multiple analytical platforms—including ELISA, radioimmunoassay, microparticle enzyme immunoassay, and the latex immunoturbidimetric method employed here [11, 12, 14, 16–19]. Results for CSF β2-M are broadly comparable across different technical platforms. This consistency therefore attests to its reliability for multiple centers.

Despite emerging evidence supporting CSF IL-10 as a promising biomarker for CNSL, its quantification, unlike CSF β2-M, suffers from substantial methodological variability, with reported thresholds ranging from 3 to 21.77 pg/mL for CNSL across ELISA, electrochemiluminescence immunoassay, and cytometric bead array platforms [12, 20–22]. It is noteworthy that common IL-10 detection methods, such as ELISA and bead-based multiplex immunoassays, are less routinely available than the immunoturbidimetric assays used for β2-M. Furthermore, these techniques often require specialized instrumentation, prolonged turnaround times, and incur higher costs. In contrast, β2-M detection via latex immunoturbidimetric assays is technically straightforward, cost-effective, and highly reproducible [23]. In our cohort, combining CSF β2-M with IL-10 significantly improved diagnostic performance over IL-10 alone, underscoring the value of a multi-marker strategy.

Our data reveal a fundamental distinction in β2-M dynamics between CNSL and NCNSL. In NCNSL, the pattern was consistent with passive diffusion across the blood–brain barrier (BBB). CSF β2-M levels were lower than serum levels, and the two compartments exhibited a moderate positive correlation. In contrast, CNSL patients displayed a pronounced dissociation, characterized by CSF β2-M concentrations approximately twice those in serum, with no significant correlation between the compartments. This dissociation suggests that mechanisms beyond simple passive diffusion are likely operative in CNSL. The compromised BBB in CNSL could facilitate the entry of small proteins like β2-M into the CSF [4, 24]. However, the complete lack of correlation between CSF and serum levels, combined with comparable CSF concentrations in PCNSL and SCNSL despite differing serum levels, argues against serum-derived diffusion as the sole source. Therefore, while a contribution from BBB disruption cannot be excluded, our findings suggest that local production by infiltrating malignant or immune cells within the CNS may also contribute to the elevated CSF β2-M levels observed in CNSL.

Longitudinal monitoring of CSF β2-M revealed dynamic level changes that closely tracked clinical disease activity, suggesting its potential utility as a monitoring tool. In complete responders, CSF β2-M levels decreased substantially following treatment, whereas levels remained elevated in those with progressive disease. Among the relapsed cohort, CSF β2-M rose markedly from remission to relapse and trended downward upon retreatment. It should be noted that the inclusion of both partial and complete responders in this group may have influenced certain statistical comparisons. Despite the constraints of a limited sample size, this trend aligns with historical reports of β2-M’s prognostic value [18, 19, 25]. For future clinical adoption, prospective validation with standardized serial sampling is required.

This study has limitations. The similar elevations of CSF β2-M observed in both CNSL and CNS infectious diseases in our cohort limit its diagnostic specificity to some extent. The retrospective single-center design may introduce selection bias. Subtype analyses (e.g., T-cell, NK/T-cell, B-cell lymphoma) were precluded by small sample sizes. Furthermore, there is an absence of data on circulating tumor DNA, a promising biomarker for CNSL with the capacity for highly sensitive and specific diagnosis, molecular characterization, and treatment monitoring [26–28].

## Conclusion

CSF β2-M remains a crucial biomarker for CNS lymphomas, supported by decades of global research and methodological consistency. Its cost-effectiveness, rapid detection, and diagnostic utility position it as an indispensable tool. Future prospective multicenter studies and combinations of multiple biomarkers are needed to validate these findings and optimize early intervention strategies for patients with CNS lymphoma.

## Data Availability

The raw data are available upon reasonable request.
